# Clinical Utility of Synthesized 18-Lead Electrocardiography

**DOI:** 10.3390/s24185947

**Published:** 2024-09-13

**Authors:** Tetsushi Yamamoto, Hiroyuki Awano, Shuichiro Ogawa, Masafumi Matsuo

**Affiliations:** 1 Nagahama Institute of Bio-Science and Technology, Nagahama 526-0829, Japan; s_ogawa@nagahama-i-bio.ac.jp; 2 Research Initiative Center, Organization for Research Initiative and Promotion, Tottori University, Yonago 683-8503, Japan; awano@tottori-u.ac.jp; 3 Graduate School of Science, Technology and Innovation, Kobe University, Kobe 657-8501, Japan; matsuo@kobe-u.ac.jp

**Keywords:** synthesized 18-lead electrocardiography, acute coronary syndromes, arrhythmias, acute pulmonary embolism, Duchenne muscular dystrophy

## Abstract

Eighteen-lead electrocardiography (18-ECG) includes, in addition to those in standard 12-lead ECG (12-ECG), six additional chest leads: V7–V9 and V3RV5R. Leads V7–V9 require the patient to be in a lateral decubitus position for the electrodes to be attached to the back. Synthesized 18-ECG (syn18-ECG) is a method that only records 12-ECG and uses computational logic to record the posterior wall (V7–V9) and right-sided (V3R–V5R) leads. We review the clinical utility of syn18-ECG in conditions including acute coronary syndromes, arrhythmias, acute pulmonary embolism, and Duchenne muscular dystrophy. The syn18-ECG waveform correlates well with the actual 18-ECG waveform, indicating that syn18-ECG is an excellent substitute for 18-ECG, excluding negative T waves. ST elevation in leads V7–V9 has the effect of reducing missed acute coronary syndromes in the posterior wall. In cases of arrhythmia, syn18-ECG can accurately estimate the target site of radiofrequency catheter ablation using a simple algorithm. The use of additional leads in Duchenne muscular dystrophy is expected to provide new insights. To facilitate gaining more knowledge regarding diseases that have not yet been investigated, it is imperative that the cost of syn18-ECG is reduced in the future.

## 1. Introduction

### 1.1. Aim of This Review

Eighteen-lead electrocardiography (18-ECG) provides more useful information than 12-lead electrocardiography (12-ECG) in many diseases, as indicated by the AHA [1]. The technology for synthesized 18-ECG (syn18-ECG), a simple synthesis of 18-ECG, was reported in 2003 [2]. Despite the technology being in existence for approximately 20 years, there have been few reports from clinical practice using syn18-ECG. Therefore, this review aims to reaffirm the clinical usefulness of 18-ECG, identify its limitations, and discuss its current standing and future in clinical practice.

### 1.2. Methods

We searched PubMed for articles published from 2003 to date using the keyword “synthesize 18-lead electrocardiography”. The search returned 20 articles. Of these, 10 articles were specifically regarding syn18-ECG. We have summarized all 10 articles.

### 1.3. 18-Lead Electrocardiography

**Figure 1:**
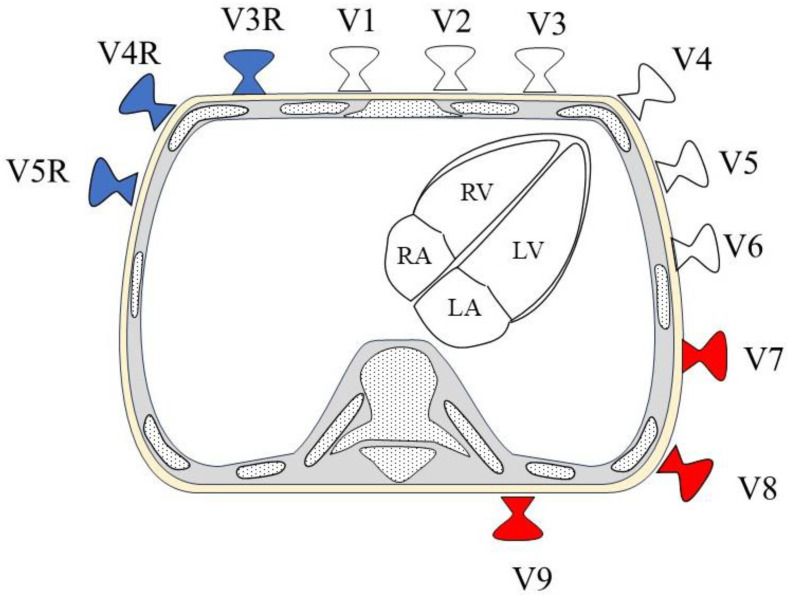
Positioning of chest electrodes and heart in 18-ECG chest electrode in horizontal cross-section. White: Electrodes of a standard 12-ECG; Red and blue: Electrodes added in 18-ECG; LV: left ventricular; LA: left atrium; RV: right ventricular; RA: right atrium.

**Figure 2:**
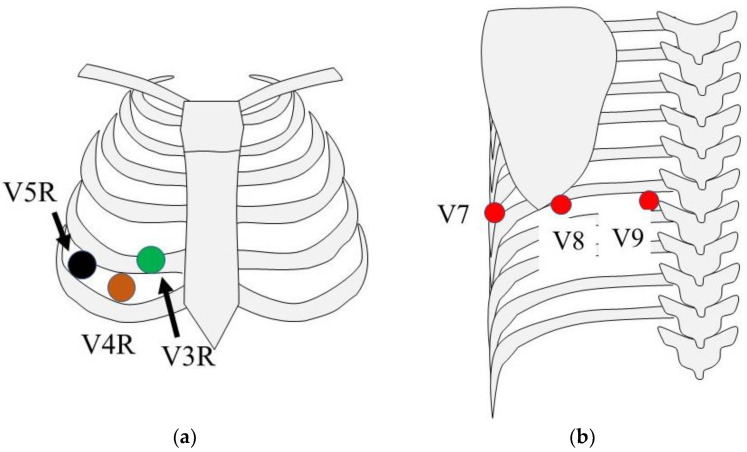
Additional chest electrode attachment sites: (**a**) precordial lead site; (**b**) electrode position on the left rear section.

Eighteen-ECG includes, in addition to those in standard 12-ECG, six additional chest leads: V7–V9 and V3R–V5R (Table 1). Eighteen-ECG provides more right ventricular information at V3R–V5R and left ventricular posterior wall information at V7–V9 than 12-ECG because of the additional electrodes (Figure 1). In contrast, 12-ECG is generally recorded in the supine position with six chest electrodes attached and at rest. When actual 18-ECG is recorded using common 12-ECG, six additional chest lead electrodes must be reattached after standard 12-ECG is recorded because of the lack of chest lead electrodes. For V3R to V5R, electrodes are attached at the symmetrical sites of V3 to V5 centered on the sternum (Figure 2a). V7 to V9 leads are attached on the back and require repositioning the patient from a supine to a left lateral decubitus position to reattach the leads (Figure 2b). The patient is then returned from the left lateral decubitus to the supine position, and the ECG is recorded. In our experience, an 18-ECG requires only about 5 min longer than a 12-ECG. However, because there is no income support in Japan, 18-ECG is not widely used due to the additional time and effort required, despite its usefulness in obtaining right and left ventricular posterior information (Table 1). Syn18-ECG addresses these problems (Table 2).

The syn18-ECG method only records a 12-ECG and uses computational logic to record the posterior wall (V7 to V9: red) and right-sided leads (V3R to V5R: blue). Additional chest electrodes are automatically placed on the whole heart by computational logic.

From V3R to V5R, electrodes are attached at symmetrical sites corresponding to V3 to V5, centered on the sternum. V7–V9 are attached at the same trunk level as V4, intersecting the posterior subaxillary line, the left mid-scapular line, and the left margin of the spine, respectively.

### 1.4. Syn18-ECG

Syn18-ECG records 12-ECG and uses computational logic to capture the posterior wall (from V7 to V9) and right-sided leads (from V3R to V5R) [2]. The principle of syn-18 ECG is to estimate the instantaneous heart vector from 12-ECG and create synthesized leads through matrix calculations of each coefficient [2,3]. Thus, V7–V9 can be recorded without reattaching the chest electrodes and moving the patient to a lateral decubitus position. Syn18-ECG is, therefore, cost-effective in terms of time and effort compared to the actual 18-ECG (Table 2). The principle of syn18-ECG was reported in 2003 and has since been applied clinically [2]. However, the limited number of facilities offering syn18-ECG and the high cost of the machine have prevented its widespread use.

This article reviewed studies using syn18-ECG and summarized its clinical utility in patients with acute coronary syndromes (ACS), arrhythmias, acute pulmonary embolism, and Duchenne muscular dystrophy (Table 3) [3,4,5,6,7,8,9,10,11,12,13].

## 2. Syn18-ECG and Actual 18-ECG Are Equivalent

Syn18-ECG is obtained by computational logic from 12-ECG and should be equivalent to the actual 18-ECG recorded. Previously, in a total of 30 participants (25 male and 5 female) with chest pain syndrome, the P-wave, QRS wave, T-wave height, and duration of syn18-ECG were consistent with those of actual 18-ECG [4]. In a study of 16 patients with ACS [4] and 56 patients undergoing catheterization [14], ST elevation on syn18-ECG was consistent with ST elevation on actual 18-ECG. In 33 patients with ST elevation ACS in whom the site of ischemia was confirmed by coronary angiography, a syn18-ECG showed ST elevation consistent with the site of ischemia on coronary angiography [5]. These results show that the syn18-ECG waveform is consistent with the 18-ECG waveform, indicating that syn18-ECG is an excellent substitute for 18-ECG.

## 3. Acute Coronary Syndromes

Patients with ACS often complain about chest pain, and it is extremely important to distinguish ACS from other chest pain diseases, such as aortic dissection and pulmonary embolism. ECG is an important early differential test for ACS. ACS with the right coronary artery or circumflex stenosis is difficult to diagnose using 12-ECG in some cases [15]. In ACS of the posterior wall, ST elevation is seen in V7–V9 [16,17,18,19,20,21]. Diagnosis may be difficult because of little ST elevation on 12-ECG. In addition, some cases of ACS in the right coronary artery territory with right ventricular infarction do not show ST elevation on 12-ECG [22,23,24]. Such cases may be misdiagnosed with a 12-ECG. In contrast, syn18-ECG automatically records V7–V9 and V3R–V5R, making it possible to detect ST elevation in the right coronary artery region and left ventricular posterior wall, simplifying accurate diagnosis [5]. Eighteen-ECG has been reported to increase the diagnostic sensitivity of ACS by 8% to 22% compared with 12-ECG [22,23,24].

## 4. Arrhythmias

Cases of lethal arrhythmias and frequent tachycardia worsen patient prognosis [25,26,27]. Radiofrequency catheter ablation is the standard therapy for such cases. Predicting the site of origin before radiofrequency catheter ablation is important for smooth radiofrequency catheter ablation. Tachyarrhythmias are classified as ventricular or atrial in origin. The algorithms used to differentiate the site of origin of these arrhythmias on a 12-ECG are complicated and often lead to misdiagnosis. Clinicians estimate the location of the accessory pathway from the waveform of V1 in Wolff–Parkinson–White syndrome, the target disease for ablation, but there are many missed leads [8].

When the site of ventricular tachycardia was predicted in patients using V3R to V5R of syn18-ECG, biphasic RS pattern in V5R could determine ventricular tachycardia in the right ventricular outflow tract with a sensitivity of 87% and specificity of 91% [10]. Comparison of R and S wave heights in V3R–V5R showed that ventricular tachycardia originating from the left ventricular outflow tract, right ventricular outflow tract septum, and right ventricular outflow tract free wall could be classified with 100% sensitivity and 100% specificity, 85% sensitivity and 100% specificity, and 100% sensitivity and 85% specificity, respectively [3]. As described above, V3R–V5R of syn18-ECG can be used to predict the origin of ventricular tachycardia with high accuracy using simple algorithms.

In atrial tachycardia, it has been reported that syn18-ECG is useful in identifying the site of origin [9]. Atrial tachycardia originates from three locations: near the superior vena cava, the right superior pulmonary vein, and the right inferior pulmonary vein [28,29]. Atrial tachycardia is treated with radiofrequency catheter ablation as ventricular tachycardia. Although studies use the P wave of a 12-ECG to identify the site of origin, the method using a 12-ECG cannot classify the three types of atrial tachycardia [30,31,32]. The P wave of V7 in syn18-ECG can classify atrial tachycardia of the right inferior pulmonary vein origin and the other two atrial tachycardias with a sensitivity of 97% and specificity of 90% [9].

Diagnosis using only V1 on a 12-ECG may misdiagnose the location of the accessory pathway in Wolff–Parkinson–White syndrome. It is particularly difficult to distinguish between types with an accessory pathway in the septum and those with an accessory pathway in the right ventricle. Nakano et al. reported the accuracy of determining the location of the accessory pathway using V1 of a 12-ECG or by adding V3R to V5R of a syn18-ECG in 44 patients in whom the location of the accessory pathway had been determined by radiofrequency catheter ablation [8]. V1, V3R, and V5R were misdiagnosed in cases with septal accessory pathways, but QS or Qr in V4R was 100% diagnosed [8].

## 5. Acute Pulmonary Embolism

Acute pulmonary embolism is an embolization of pulmonary capillaries caused by a thrombus or other embolus in the pulmonary vessels. Acute pulmonary embolism has a fatal outcome and must be diagnosed quickly and accurately [33]. The standard 12-lead electrocardiographic findings of acute pulmonary embolism are right-axis deviation, S waves in lead I, Q waves in lead III, and negative T waves in lead III [34]. Fifty-six patients diagnosed with acute pulmonary embolism using contrast-enhanced CT were analyzed for 12-ECG and V3R to V5R ECG findings in syn18-ECG. Multivariate logistic regression analysis revealed that only the negative T wave of V3R was an independent determinant of pulmonary embolism [11].

## 6. Duchenne Muscular Dystrophy

Duchenne muscular dystrophy (DMD) is a progressive muscle disease that occurs in 1 of 5000 live births [35] with an average life expectancy of about 30 years [36,37,38]. The most common cause of death in this condition is cardiac-related [39], and about half of all cardiac dysfunction occurs at around 14 years of age [40]. The latest DMD treatment guidelines recommend that echocardiography be performed once a year for patients aged 6–12 years and twice a year for those aged 12 and older [41]. However, echocardiography cannot be performed as frequently as ECG because of the difficulty in obtaining images due to scoliosis and noninvasive positive pressure ventilation placement. In contrast, ECG testing can be easily performed in many laboratories. Many types of abnormal findings have been reported in patients with DMD using 12-ECG [42,43,44,45]. Cardiac dysfunction in DMD originates in the left ventricular posterior wall [46,47,48,49]. Recently, ECG abnormalities in DMD have been considered unrelated to cardiac dysfunction [50]. The reason for this is that the studies to date have been 12-ECG studies without electrodes on the left ventricular posterior wall. However, patients with DMD require even more effort and time than usual to attach electrodes to the back because the body is immobile. Therefore, we investigated the decrease in R wave amplitude in 193 patients with DMD in detail using syn18-ECG. R wave amplitude was found to decrease at V6 to V9, a greater and more rapid decrease than the usual decrease, approximately 2 years before the cardiac dysfunction [12]. Without seeing the lead from V7 to V9, the clinician might not notice the sharp decrease in amplitude in V6. A sharp decrease in R wave amplitude is one of the indicators for starting medical treatment for DMD. In the general hospital setting, cardiac dysfunction in DMD can be predicted using a 12-ECG to check for a sharp decrease in the R wave amplitude of the V6 lead.

## 7. Limitations

However, we must be cautious when evaluating negative T waves with syn18-ECG. In a study of 295 chest pain subjects, negative T waves observed in 14 subjects on actual 18-ECG were absent on syn18-ECG, and conversely, negative T waves that should have been absent in three subjects were present on syn18-ECG [6]. However, there are no papers that examine the cause. It is hypothesized that the displacement may be due to an extremely thin chest wall or myocardium [6]. The syn18-ECG algorithm assumes that 12-lead ECG is accurately attached. Therefore, the actual 18-ECG may differ if there is a physical deformation of the thorax, such as scoliosis, or if the heart is in a different position from the usual after pneumonectomy. Furthermore, extremely abnormal ECGs have not been studied and may deviate from this algorithm. Great care should be taken when using syn18-ECG in such cases.

## 8. Conclusions

No previous studies have reviewed syn18-ECG to date. Syn18-ECG is 18-ECG obtained from a 12-ECG recording. Syn18-ECG is cost-effective and easy to produce, although it is more expensive than standard 12-ECG. In this review, the usefulness of syn18-ECG was evaluated based on published data, and it was found to be similar to that of actual 18-ECG. To facilitate gaining more knowledge regarding diseases that have not yet been investigated, it is imperative that the cost of syn18-ECG is reduced in the future. The use of 18-ECG for more accurate diagnosis of various diseases is recommended as syn-18 is a powerful tool for this purpose.

## Figures and Tables

**Table 1:** Additional information obtained from 18-ECG.

	12-ECG	18-ECG
Additional chest electrode		V3R–V5R and V7–V9
Chest electrode for evaluation of the right ventricle	V1–V2	In addition to left, V3R–V5R
Chest electrode for evaluation of the left ventricular posterior	Nothing	V7–V9

**Table 2:** Comparison of actual 18-ECG and syn18-ECG using 12-lead electrocardiograph.

	Actual 18-ECG	syn18-ECG
Reattachment	Necessary	Not necessary
Lateral decubitus	Necessary	Not necessary
Additional recording time after 12-ECG recording	Necessary	Not necessary
Electrocardiograph used	Most common electrocardiograph	Dedicated special electrocardiograph
Waveform comparison	Measured waveform	Synthesized waveform

**Table 3:** Usefulness of syn18-ECG.

	12-ECG	syn18-ECG
Diagnosis of acute coronary syndrome		
Right ventricular	Little ST elevation	ST elevation in V3R–V5R
Left ventricular posterior	Little ST elevation	ST elevation in V7–V9
Diagnosis of arrhythmia		
Accessory pathway in the septum of the WPW	Difficulty	QS or Qr in V4R
Atrial tachycardia of right inferior pulmonary vein origin	Difficulty	Isoelectric P wave in V7
Others		
Diagnosis of acute pulmonary embolism	Low sensitivity and nonspecificity	Negative T wave in V3R
Cardiac dysfunction in Duchenne muscular dystrophy	Not related	Sharp decrease R amplitude in V7–V9

## Data Availability

The data presented in this study are available on request from the corresponding author.
